# Association analysis of *SOCS3*, *JAK2* and *STAT3* gene polymorphisms and genetic susceptibility to type 2 diabetes mellitus in Chinese population

**DOI:** 10.1186/s13098-021-00774-w

**Published:** 2022-01-06

**Authors:** Yang Zhang, Chunwen Lin, Rong Chen, Ling Luo, Jialu Huang, Hao Liu, Weiying Chen, Jian Xu, Haibing Yu, Yuanlin Ding

**Affiliations:** 1 Department of Epidemiology and Medical Statistics, School of Public Health, Guangdong Medical University, Dongguan, China; 2 Affiliated Hospital of Guangdong Medical University, Zhanjiang, China

**Keywords:** Suppressor of cytokine signaling 3 (*SOCS3*), Janus kinase 2 *(JAK2*), Signal transducer and activator of transcription 3 (*STAT3*), Gene polymorphisms, Type 2 diabetes mellitus (T2DM)

## Abstract

**Aim:**

The association of polymorphisms in the three genes of SOCS3, JAK2 and STAT3 with genetic susceptibility to type 2 diabetes mellitus (T2DM) was explored, and its interaction with environmental factors such as hypertension and triglycerides was analyzed.

**Methods:**

The Hardy–Weinberg balance test was used to analyze the random balance of genes in the population. The analysis of the association of SNPs with T2DM was performed using Pearson’s chi-square test. Haplotype frequency distribution, SNPs-SNPs interaction and environmental factors were analyzed by chi-square test and logistic regression.

**Results:**

The genotype distribution of SNPs rs2280148 of the SOCS3 gene was statistically significant. The allele frequency distribution of SNPs (rs4969168/rs2280148) was statistically different. After covariate correction, the SOCS3 gene locus (rs4969168) showed an association with T2DM in additive model, while the rs2280148 locus showed an association with T2DM in all three models. The locus (rs10974914/rs10815157) allele and genotype frequency distribution of JAK2 were statistically significant. After covariate correction, two SNPs in the gene showed association with T2DM in both additive and recessive models. The distribution of genotype frequencies of SNPs rs1053005 locus in gene STAT3 was statistically significant between the two groups. In recessive genetic models, rs1053005 locus polymorphisms was associated with T2DM. Haplotype S3 (G G)/S 4 (G T) of the SOCS3 gene as well as haplotype J2 (A G)/J 3 (G C) of the JAK2 gene were closely associated with T2DM. There was an interaction between SNPs rs4969168 and SNPs rs2280148 in the SOCS3 gene. There was an interaction between the SOCS3, JAK2 and STAT3 genes and hypertension/triglycerides.

**Conclusion:**

The SOCS3 and JAK2 genes may be associated with T2DM in the Chinese population, in which SNPs carrying the A allele (rs4969168)/G allele (rs2280148)/C allele (rs10815157) have a reduced risk of T2DM. Haplotype S3 (G G)/S 4 (G T) of the SOCS3 gene and haplotype J2 (A G)/J 3 (G C) of the JAK2 gene may be influencing factor for T2DM. The interaction between SNPs rs4969168 and SNPs rs2280148 increases the risk of T2DM. Hypertension and triglycerides may interact with SNPs of T2DM susceptibility genes.

## Introduction

Diabetes has quadrupled in the past 30 years and is the ninth leading cause of death [1]. Currently, approximately 1 in 11 adults (20–79 years) worldwide have diabetes (463 million people), and 90% of them have type 2 diabetes mellitus (T2DM) [2]. Diabetes is expected to become the seventh leading cause of death in developing countries by 2030 due to a rapid rise in the incidence of diabetes [3]. The epidemic of T2DM is mainly found in Asia, and is most pronounced in countries with middle economic level, such as China [2, 3]. It is one of the major non-communicable health problems in the world [4]. T2DM is mainly manifested as the deficiency of the body’s own islet function or insulin dysfunction, which is a multi-factor disease with the joint action of genetic factors and environmental factors [5–7]. Genetic susceptibility is one of the main influencing factors of T2DM, which is characterized by a higher concordance rate in identical twins and racial differences in the familial aggregation [8–10]. Genetic susceptibility to T2DM has led to an intensive study for nearly past few decades. However, the specific genetic architecture of T2DM risk and the underlying mechanism by which the risk gene of T2DM remains poorly understood. Symptoms of T2DM are often accompanied by inflammatory responses [11]. In the inflammatory response, the JAK/STAT signaling pathway plays an important role and affects the insulin signaling pathway under the SOCS3 (suppressor of cytokine signaling3) gene. Therefore, we hypothesized that the SOCS3, JAK2(Janus kinase 2), and STAT3 (Signal transducer and activator of transcription 3) genes involved in the JAK/STAT signaling pathway may have an association for genetic susceptibility to T2DM.

SOCS3 is a cellular signaling inhibitory protein with a relative molecular mass of 24.7 kd, consisting of 225 amino acids. It plays an important role in cell signaling, with functions such as regulating cell physiological processes, regulating inflammation, inhibiting proliferation of cancer cells and metastasis [12]. Recent studies have found that the overexpression of SOCS3 is closely associated with insulin resistance and blood glucose levels in diabetic patients. SOCS3 as a inhibitory factor of insulin signaling interacts with the insulin receptor or induces the degradation of signaling proteins that in turn suppresses the conduction of insulin signaling [13, 14]. At the same time, it can lead to insulin resistance by participating in the conduction processes of other cytokines (TNF-α, IL6, and PPAR) signaling pathways [14]. Insulin can affect *β* cell function, enhance insulin gene transcription, and promote or inhibit *β* cell apoptosis [15]. In *β* cells, the SOCS3 gene is able to regulate insulin levels by regulating leptin and inhibiting the conduction of JAK/STAT signal channel [16]. Thus, we speculate that the SOCS3 gene may be a risk factor for T2DM.

The JAK2 and STAT3 genes are key genes in the JAK/STAT signaling pathway. The molecule of JAK2 contains two kinase domains: the C-terminal domain contains the structures associated with the tyrosine protein kinase and has the catalytic activity, while the N-end domain lacks the amino acids associated with the catalytic phosphorylation reactions and thus has no tyrosine kinase activity [17]. The JAK2 gene polymorphisms was found to be closely associated with the metabolic syndrome in T2DM and related complications [18]. Chen et al. [19] found that the overexpressed JAK2 gene can increase the activity of the insulin promoter, with opposite effects when the JAK2 gene is silenced or poorly expressed. Amyloid-*β* protein can lead to insulin resistance as well as the development of T2DM through the activation of the JAK2/STAT3/SOCS1 pathway [20]. We also found that Lep-STAT3 signaling pathway is closely associated with metabolic diseases such as hyperglycemia, hyperinsulinemia and glucose tolerance [21]. It has been found that the JAK2 and STAT3 genes play an important role in the pathogenesis of T2DM by participating in the JAK/STAT signaling pathway.

T2DM is a complex disease controlled by polygenes, and environmental factors also have an important role in the pathogenesis [22]. Simply analyzing the association of only a single gene or individual environmental factors with T2DM often leads to the neglect of some factors with weak effects. Therefore, we analyzed the association of the three genes (SOCS3/JAK2/STAT3) and their SNPs-SNPs interaction with T2DM in the Chinese Guangdong population and analyzed the role of environmental factors involved in it. The aim was to explore the relationship between genetic polymorphisms in SOCS3, JAK2 and STAT3 and the genetic susceptibility to T2DM.

## Materials and methods

### Study population

This study was a case control study. In 2013, 599 T2DM patients (30–70 years) were recruited from endocrinology departments of eight hospitals in Guangdong Province, China. A total of 677 healthy subjects who were enrolled in the same physical examination centers of the 8 hospitals were selected as the normal control (NC) group. Both groups were Han people in Guangdong province and had no genetic relationship. The consistency of genetic background is high in the population. The project has been approved by the Ethics committee. All subjects were been informed of the specific study background and signed informed consent. We used the T2DM diagnostic criteria of Diabetes proposed by American Diabetes Association (ADA): (1) HbA1c (glycated hemoglobin A1c) ≥ 6.5%; (2) FPG (fasting blood-glucose) ≥ 7.0 mmol/L; and (3) Blood glucose was 2 h after glucose load ≥ 11.1 mmol. Control group inclusion criteria: (1) Age 30–70; (2) No family history of diabetes; (3) No other serious diseases, such as cardiovascular and cerebrovascular diseases; and (4) No abnormalities in biochemical results such as physical examination and blood glucose test.

### Information inquiry

Subjects who met the inclusion criteria were given a questionnaire and physical examination. A survey scale suitable for the purpose of the study was designed. The survey mainly includes four aspects: demographic information, lifestyle information, clinical examination data and history of drug taking. Standardized questionnaire survey was conducted by uniformly trained investigators, including general information (age, height, weight, sex, native place and occupation, etc.) and medical history (disease history, disease course, smoking history, family history, complications, diet and exercise status, etc.). Calculate Body Mass Index [BMI = weight (kg)/height (m^2^)]. The blood pressure of the right arm was measured with a desktop mercury sphygmomanometer twice at an interval of 10 min, and the average value was taken. The diagnostic criteria for hypertension were referred to the 1999WHO/ISH guidelines for hypertension: (1) Systolic blood pressure (SBP ≥ 140 mmHg); and (2) Diastolic blood pressure (DBP ≥ 90 mmHg).

### Blood sample collection

Professional endocrinology nurses collected 9 ml of peripheral blood in the morning from all subjects with at least 8 h of fasting. 5 ml of blood samples were used to detect clinical biochemical indicators. Including triglycerides (TG), fasting blood glucose (FPG), high density lipoprotein cholesterol (HDL-C), total cholesterol (TC), low density lipoprotein cholesterol (LDL-C) and glycated hemoglobin A1c (HbA1c). Glycosylations method was used for determination of FPG level and enzyme method was used for determination of TC, TG, HDL-C and LDL-C. HbA1c was determined by high performance liquid chromatography (BIO-Radd-10 TM Hb A1c detection system). The other biochemical parameters were determined by automatic biochemical analyzer (Bio-RAD Corporation, USA).

### Extracted DNA

The remaining 4 ml blood sample was used to extract DNA. Ethylene Diamine Tetraacetic acid-K2 (EDTA-K2) was used for adequate anticoagulation treatment of blood samples. After digestion by Protease K, DNA was extracted by salting out method. Store the extract in a refrigerator at − 80℃. For specific extraction steps, we referred to the corresponding technical instructions:

1. The blood samples were transferred from the anticoagulant tube to the 10 ml centrifuge tube and marked on the centrifuge tube.
2. 2 ml cell lysis solution was added to the blood samples containing anticoagulant, and centrifuged for 10 min (5000 r/min, r = 7.1 cm) after mixing.
3. Remove the liquid from the top of the centrifuge tube, then invert it on a clean blotting paper for 1 min to dry.
4. A mixture of buffer and protease K is configured in time (1 mL buffer + 10 μL protease K solution).
5. Add 1 mL mixture to each sample and mix thoroughly.
6. Take a water bath at 65℃ for 20 min, add 1 mL isopropyl alcohol, and mix thoroughly until filamentous or clumps appear.
7. Centrifugation was performed for 15 min (5000 r/min, r = 7.1 cm), and the upper liquid was discarded.
8. Add 1 mL 70% ethyl alcohol, transfer it into a 1.5 mL centrifuge tube, centrifuge it for 10 min (8000 r/min, r = 4.2 cm), and the upper liquid was discarded. Perform two operations
9. The tube is dried at room temperature until the liquid in the DNA precipitate evaporates.
10. The corresponding elution buffer was added according to the mass of DNA, and then the mixture was bathed at 65℃ for 1 h. During this time, flick the finger several times to help blend and centrifuge for 30 s.
11. DNA quality was tested by nucleic acid content detector, and DNA concentration (A260/280 and A260/230) was read to judge the purity of DNA. Finally, DNA was stored in a refrigerator at − 80 ℃.

### SNPs selection and genotyping

We selected tagSNPs [MAF (minor allele frequency) > 0.05, R2 ≥ 0.8] from Upstream and downstream 5 kb region of target gene by Haplotype Mapping (HapMap) public database and Hapoloview (ver.4.2). TagSNPs with high prediction scores were screened by FastSNP (function analysis and selection tool for single nucleotide polymorphisms, http://fastsnp.ibms.sinica.edu.tw). Then six Tag SNPs (rs4969168/rs2280148, rs10974914/rs10815157 and rs1053005, rs957970) were selected from the three genes (SOCS3/JAK2/STAT3). The TaqMan^®^ SNPs probe method was used for typing. The basic principle of this technique is to use the high specificity of ligase chain reaction to realize the recognition of SNPs alleles. Then non-specific sequences of different lengths were introduced into the end of the ligator probe and ligase chain reaction was used to obtain the ligator products of different lengths corresponding to the loci. Universal primers labeled with fluorescence were used to PCR amplify the ligands, and electrophoresis was used to separate the amplified products. Finally, the genotypes of the loci were obtained by analysis of electrophoresis patterns. The corresponding sequences are shown in Table 1.

**Table 1:** Six sequences corresponding to the SNPs loci

SNPs	No	Needle sequences
rs10974914	C_386825_10	CTTTATTCAACAGATTT[A/G]TTAATCAACACATGTT
rs10815157	C_1417103_10	AACCGATGTAAAATCA[C/G]TTATTGCCTACTCCTCT
rs1053005	C_7530672_10	GCGAACCCTGTTCATC[C/T]TAGAGAAGGTCGTCTC
rs957970	C_1952200_10	AAGTTTTTCAAAAATCT[A/G]GAACACACTTTTAGC
rs4969168	C_1952200_10	AGCTGACCAGCCCATCC[A/G]TCCCCTCCAAATGTT
rs2280148	C_15969511_10	GGGGCCTGTCCGCCCTC[G/T]TTCCCCCCAGAGCTA

The specific steps are as follows:

1. Items prepared: crushed ice, DNA samples and typing reagents. (Genotype Assay* and Universal PCR Master Mix*).
2. The DNA samples were defrosted at low temperatures. Combine the mixture in proportion. (20 × Genotyping assay = 0.25*N*1.1 μl, 2 × universal PCR Master Mix = 2.5*N*1.1 μl, ddH2O = 4.5*N *1.1 μl).
3. Add mixture (4.5 μl) and DNA (1-20 ng) to Orifice.
4. Design allele typing template (AD file) and PCR amplification template (AQ file). The PCR reaction condition was 95℃ for 10 min. 40 PCR cycles were performed: denaturation at 92 °C for 15 s, annealing at 60 °C for 1 min.
5. Raw data were collected and analyzed on a 7900HT fluorescent quantitative PCR instrument. The fluorescence labeling curves of PCR products and the corresponding SNPs locus/allele information were obtained.

*Reagents purchased from Applied Biosystems.

In the typing test, 10% of the samples were randomly selected from the case group and the control group respectively, and the experiment was repeated. The results of the two typing experiments were compared, and the false positive rate was less than 5%, indicating that the results were reliable.

### Statistical analysis

Quantitative measurement data were expressed as mean ± standard deviation, and qualitative data were expressed as case number or percentage. Student’s *t*-test was used to analyze the difference between the two groups of continuous quantitative data. Genotypes and allele frequencies were compared by Chi-square test. The *HWE* (Hardy–Weinberg equilibrium) test of genotype distribution was performed by *χ2* test of goodness of fit. LD block haplotype analysis was performed by unconditional Logistic regression analysis. Allele association analysis, genotype association analysis and Cochran-Armitage trend test were used to analyze the relationship between SNPs and T2DM. Haplotype frequency distribution, SNPs-SNPs interaction, and environmental factors were analyzed by chi-square test and logistic regression. *P*-value < 0.05 was considered as statistical significance.

## Results

### Baseline data

In order to prevent a small number of subjects with poor genotyping results from bias to the overall analysis, individuals with a loss rate of over 20% SNPs genotyping were excluded. Finally, there were 599 cases and 677 normal controls (NC) selected for further study. Among them, there were 304 male (50.75%) in T2DM group, with the average age of 58.23 years, while there were 424 male (62.23%) in NC group, with the average age of 58.12 years. The BMI, FPG and TG of NC group were lower than those of T2DM group (*P* < 0. 001). The baseline data is shown in Table 2.

**Table 2:** Comparison of general data and biochemical indexes between the two groups

Group	N (male/female)	Age (years)	BMI (kg/m2)	Hypertension	FPG (mmol/L)	TG (mmol/L)	TC (mmol/L)	HDL-C (mmol/L)	LDL-C (mmol/L)
NC	677 (424/253)	58.12 ± 10.42	23.39 ± 3.29	258 (41.5%)	5.40 ± 0.97	1.36 ± 1.02	5.41 ± 1.05	1.37 ± 0.38	3.05 ± 0.69
T2DM	599 (304/295)	58.23 ± 10.57	24.472 ± 3.86	192 (38.6%)	10.73 ± 4.69	2.23 ± 2.65	5.36 ± 1.08	1.42 ± 0.73	2.88 ± 1.10
*P*	< 0.001	0.847	< 0. 001	0.903	< 0. 001	< 0. 001	0.536	0.328	0.031

All values were reported as mean ± SD or percentage

NC, normal control; FBG, fasting blood glucose; TC, total cholesterol; HDL-C, high density lipoprotein cholesterol; LDL-C, low density lipoprotein cholesterol; BMI, body mass index

### Analyzing of the allele and genotype frequency

Six candidate SNPs loci in the three genes were tested by Hardy-Winberg equilibrium test (*α* = 0.01). The rs957970 locus was removed (*P* < 0.001). The remaining loci indicate that the frequency distribution of genotype in the object population conforms to the law of genetic balance and is representative of the population. See Table 3.

**Table 3:** Results of Hardy–Winberg equilibrium test

Gene	SNPs	Allele		MAF	*P*_*hwe*_
		Minor	Major		
*SOCS3*	rs4969168	A	G	0.414	0.755
	rs2280148	G	T	0.217	0.614
*JAK2*	rs10974914	A	G	0.345	0.040
	rs10815157	C	G	0.347	0.033
*STAT3*	rs1053005	C	T	0.402	0.011
	rs957970	A	G	0.461	< 0.001

MAF, minor allele frequency; SNPs, single nucleotide polymorphisms; *HWE*, Hardy–Weinberg equilibrium

The allele/genotype distribution of the T2DM patients and normal persons is showed in Tables 4, 5. Table 4 showed that the allelic distribution of rs4969168, rs2280148, rs10974914 and rs10815157 were statistically significant in the two groups (*P* < 0.05). After adjusting for covariates (age, sex, BMI), the difference between the two groups was still statistically significant (P < 0.05). Chi-square test was performed for two loci in SOCS3, JAK2 and STAT3 genes. Table 5 shows that three candidate genes may be associated with T2DM.

**Table 4:** The results of association analysis between alleles and T2DM

Gene	SNPs	Allele	NC	T2DM	*P*		*OR* (95% *CI*)
					Observed	Adjust	
*SOCS3*	rs4969168	*A/G*	462/598	435/671	0.043	0.045	0.84 (0.71–1.00)
	rs2280148	*G/T*	334/1000	198/916	< 0.001	< 0.001	0.65 (0.53–0.79)
*JAK2*	rs10974914	*A/G*	407/865	415/695	0.007	0.006	1.27 (1.07–1.50)
	rs10815157	*C/G*	366/578	346/762	0.001	< 0.001	0.72 (0.60–0.86)
*STAT3*	rs1053005	*A/G*	416/646	460/656	0.311	0.330	1.09 (0.92–1.29)

OR, odds ratio

**Table 5:** The results of association analysis between genotype and T2DM

Gene	SNPs	Genotype	NC	T2DM	*P*
*SOCS3*	rs4969168	*AA/AG/GG*	98/266/166	85/265/203	0.126
	rs2280148	*GG/TG/TT*	42/250/375	19/160/378	< 0.001
*JAK2*	rs10974914	*AA/AG/GG*	61/285/290	97/221/237	< 0.001
	rs10815157	*CC/CG/GG*	91/184/197	48/250/256	< 0.001
*STAT3*	rs1053005	*CC/CT/TT*	58/300/173	98/264/196	0.001

### Genetic model analysis

To further confirm the relationship between polymorphisms and T2DM at each gene locus, we performed a univariate logistic regression analysis under different genetic models (additive model, dominant model, and recessive model). Suppose A and a are two alleles at the locus, and A is the risk allele. The corresponding genotypes were AA, Aa and aa, and the sample size was N. Allele frequency calculation of each gene: the frequency of A gene is P(A) = (NAa + 2NAA)/2 N. The association between SNP alleles or genotypes and T2DM was analyzed based on additive, dominant and recessive gene models. Genotype corresponding encoding (additive model: AA = 2, Aa = 1, aa = 0; Dominant model: AA = 1, AA = 1, AA = 0; The implicit model is AA = 1, AA = 0, AA = 0).

To make the analysis results more reliable, four covariates (age, sex, BMI) were adjusted under three models. Where locus rs2280148 was statistically different in three models, while locus rs4969168 associated with T2DM in additive and dominant models. Two SNPs loci (rs10974914 and rs10815157) in the JAK2 gene were significantly associated with T2DM in the additive and recessive models (*P* < 0.05). In the recessive model, the rs105300 locus was associated with T2DM [*P* = 0.002, *OR* (95% CI) = 1.91(1.28–2.86)], as detailed in Table 6.

**Table 6:** Comparison of genetic models between T2DM and NC

SNPs	Additive model				Dominant model				Recessive model			
	*P*	*OR* (95%* CI*)	*adP*	*adOR *(95%* CI*)	*P*	*OR *(95%* CI*)	*adP*	*adOR *(95%* CI*)	*P*	*OR *(95% *CI*)	*adP*	*adOR *(95% *CI*)
rs4969168	0.044	0.84 (0.70–1.00)	0.008	0.77 (0.63–0.93)	0.062	0.79 (0.61–1.01)	0.016	0.70 (0.53–0.94)	0.171	2.00 (1.42–2.81)	0.064	0.71 (0.49–1.02)
rs2280148	< 0.01	0.65 (0.53–0.79)	< 0.01	0.61 (0.48–0.77)	< 0.01	0.61 (0.48–0.77)	< 0.01	0.56 (0.43–0.74)	0.023	0.40 (0.27–0.58)	0.048	0.51 (0.26–1.00)
rs10974914	0.007	1.25 (1.06–1.48)	0.014	1.26 (1.05–1.52)	0.316	1.13 (0.89–1.42)	0.501	1.09 (0.84–1.42)	< 0.01	1.74 (1.23–2.46)	< 0.01	2.13 (1.47–3.10)
rs10815157	< 0.01	0.73 (0.61–0.87)	0.018	0.79 (0.64–0.96)	0.151	0.83 (0.65–1.07)	0.482	0.90 (0.68–1.20)	< 0.01	0.87 (0.66–1.14)	< 0.01	0.46 (0.30–0.69)
rs1053005	0.311	1.10 (0.92–1.31)	0.119	1.18 (0.96–1.45)	0.375	0.96 (0.74–1.23)	0.865	0.98 (0.73–1.30)	0.002	0.80 (0.58–1.10)	0.002	1.91 (1.28–2.86)

Trend tests are based on predefined scores, which correspond to different genetic models. Only when the genetic model developed is the data optimal model, the corresponding trend testing effect is the greatest. The test results show the high reliability of the model analysis, and the results are detailed in Table 7.

**Table 7:** Analysis results of three robustness test methods

Gene	SNPs	MERT		MAX3		GMS	
		Z	*P*	*χ*^*2*^	*P*	*χ*^*2*^	*P*
*SOCS3*	rs4969168	− 1.991	0.046	2.020	0.091	2.020	0.072
	rs2280148	− 4.034	0.001	4.307	0.001	4.307	0.001
*JAK2*	rs10974914	3.048	0.002	4.002	0.001	4.002	0.001
	rs10815157	− 3.886	0.001	4.952	0.001	4.952	0.001
*STAT3*	rs1053005	1.393	0.164	3.126	0.004	3.126	0.004

MERT, maximin efficiency robust test; GMS, genetic model selection

### Haplotype analysis of JAK2 and STAT3 genes

The SNPs rs4969168 and SNPs rs2280148 in SOCS3 gene were constructed haplotypes, and the four haplotypes were named S1-S4. The SNPs rs10974914 and SNPs rs10815157 in JAK2 gene were constructed haplotypes, and the four haplotypes were named J1-J4. Chi-square test showed that haplotypes S3 (GG) and S4 (GT) had statistically significant differences in haplotype frequency distribution between the case group and the control group (*P* < 0.001). Same thing with J2 (AG) and J3 (GC). According to their frequency distribution, haplotypes of S3 (GG) and J3 (GC) may be the protective factors for T2DM, while haplotypes of J2 (AG) and S4 (GT) may be the risk factors for T2DM. See Table 8 for details.

**Table 8:** Results of SOCS3 and JAK2 gene haplotype analysis

	Haplotype	T2DM (%)	NC (%)	*P*	*OR* (95% *CI*)
S1	*AG*	165 (15.8)	159 (14.5)	0.414	0.91 (0.72–1.15)
S2	*AT*	294 (28.2)	271 (24.7)	0.069	0.84 (0.69–1.01)
S3	*GG*	101 (9.7)	35 (3.2)	< 0.001	0.31 (0.21–0.46)
S4	*GT*	484 (46.3)	631 (57.6)	< 0.001	1.57 (1.33–1.87)
J1	*AG*	254 (27.0)	304 (27.7)	0.707	1.04 (0.85–1.26)
J2	*AG*	32 (3.4)	98 (9.0)	< 0.001	2.85 (1.89–4.29)
J3	*GC*	109 (11.5)	36 (3.3)	< 0.001	0.26 (0.18–0.39)
J4	*GG*	547 (58.1)	656 (58.1)	0.406	1.08 (0.90–1.29)

### Analysis of SNPs-SNPs interaction among SOCS3, JAK2 and STAT3 genes

The interaction analysis between SNPs-SNPs was performed using multivariate nonconditional Logistic regression models as well as the PLINK software. The results of the analysis revealed an interaction between rs4969168 and rs2280148 in the SOCS3 gene (*P* < 0.001). This is a statistically significant SNPs-SNPs interaction pair (*α* = 0.05). After adjusting for the four covariates (age, sex, BMI), an interaction remained between rs4969168 and rs2280148 (*P* < 0.001). See Tables 9, 10 for details.

**Table 9:** The interaction of the loci between the genes

Gene	SNPs	Gene	SNPs	*OR*_IT_	*χ*^*2*^	*P*
*SOCS3*	rs4969168	*SOCS3*	rs2280148	1.997	18.61	< 0.001
*SOCS3*	rs4969168	*JAK2*	rs10974914	1.031	0.054	0.816
*SOCS3*	rs4969168	*JAK2*	rs10815157	0.984	0.013	0.910
*SOCS3*	rs4969168	*STAT3*	rs1053005	0.962	0.080	0.778
*SOCS3*	rs2280148	*JAK2*	rs10974914	1.174	1.041	0.308
*SOCS3*	rs2280148	*JAK2*	rs10815157	0.933	0.212	0.646
*SOCS3*	rs2280148	*STAT3*	rs1053005	1.003	0.0003	0.986
*JAK2*	rs10974914	*JAK2*	rs10815157	0.955	0.112	0.738
*JAK2*	rs10974914	*STAT3*	rs1053005	1.209	1.883	0.170
*JAK2*	rs10815157	*STAT3*	rs1053005	1.027	0.035	0.853

IT, interaction

**Table 10:** Interaction of each gene locus after covariate correction

Gene	SNPs	Gene	SNPs	*OR*_IT_	χ^2^	*P*
*SOCS3*	rs4969168	*SOCS3*	rs2280148	2.450	25.35	< 0.001
*SOCS3*	rs4969168	*JAK2*	rs10974914	1.018	0.016	0.900
*SOCS3*	rs4969168	*JAK2*	rs10815157	1.007	0.002	0.963
*SOCS3*	rs4969168	*STAT3*	rs1053005	0.961	0.071	0.790
*SOCS3*	rs2280148	*JAK2*	rs10974914	1.014	0.006	0.937
*SOCS3*	rs2280148	*JAK2*	rs10815157	0.863	0.058	0.089
*SOCS3*	rs2280148	*STAT3*	rs1053005	1.045	0.058	0.809
*JAK2*	rs10974914	*JAK2*	rs10815157	0.948	0.139	0.709
*JAK2*	rs10974914	*STAT3*	rs1053005	1.155	0.932	0.335
*JAK2*	rs10815157	*STAT3*	rs957970	1.02	0.020	0.889
*JAK2*	rs10815157	*STAT3*	rs1053005	1.049	0.097	0.756

### Interaction between the SNPs site and hypertension/triglycerides

The results showed that the rs10815157 locus interacts with hypertension in the recessive model [*P* = 0.03, *OR* = 0.37, 95% *CI* = (0.16–0.89)] as a protective factor for T2DM. The rs1053005 locus interacts with hypertension as a risk factor for T2DM [*P* = 0.01, *OR* = 3.23, 95% *CI* = (1.43–7.29)]. The SNPs rs4969168 locus interacts with triglycerides in the additive model, while the SNPs rs10974914 locus interacts with triglycerides in both the additive and recessive models. In the additive model, both SNPs rs4969168 and SNP rs10974914 interact with triglycerides. See Tables 11, 12 for details.

**Table 11:** Interaction analysis of SNPs with hypertension

SNPs	Additive model		Dominant model		Recessive model	
	*P*	*OR* (95% CI)	*P*	*OR *(95% CI)	*P*	*OR* (95% CI)
rs4969168	0.36	1.18 (0.83–1.69)	0.52	1.19 (0.71–1.99)	0.37	1.36 (0.70–2.63)
rs2280148	0.59	1.18 (0.65–2.13)	0.59	1.38 (0.42–4.51)	0.59	1.38 (0.42–4.51)
rs10974914	0.78	1.05 (0.75–1.48)	0.75	1.08 (0.67–1.74)	0.91	1.04 (0.51–2.13)
rs10815157	0.20	0.78 (0.53–1.14)	0.74	0.92 (0.55–1.54)	0.03	0.37 (0.16–0.89)
rs1053005	0.12	1.35 (0.93–1.96)	0.79	1.08 (0.64–1.81)	0.01	3.23 (1.43–7.29)

**Table 12:** Interaction analysis of SNPs with triglycerides

SNPs	Additive model		Dominant model		Recessive model	
	*P*	*OR* (95% CI)	*P*	*OR* (95% CI)	*P*	*OR* (95% CI)
rs4969168	0.02	1.27 (1.05–1.55)	0.04	1.33 (1.02–1.75)	0.07	1.46 (0.97–2.20)
rs2280148	0.53	1.09 (0.84–1.41)	0.53	1.18 (0.70–2.00)	0.53	1.18 (0.70–2.00)
rs10974914	0.01	0.79 (0.67–0.93)	0.06	0.78 (0.58–1.01)	0.003	0.66 (0.51–0.87)
rs10815157	0.06	1.23 (0.99–1.51)	0.18	1.22 (0.92–1.62)	0.09	1.51 (0.94–2.43)
rs1053005	0.08	1.22 (0.98–1.52)	0.045	1.33 (1.01–1.75)	0.52	1.18 (0.72–1.94)

## Discussion

T2DM is a complex disease caused by the interaction of environmental and genetic factors [23]. In complex diseases, the effect of a single gene is weak, and generally a variety of factors are involved in the occurrence and development of the disease [24]. Haplotype analysis of multiple genes and interactions between genes provide the possibility of finding such a weak effect [25]. Due to genetic heterogeneity among different populations, we selected 1276 people from Guangdong Han population to study the association between single nucleotide polymorphisms (SNPs) of gene loci and genetic susceptibility to T2DM.

This study found that the allele frequency and genotype frequency of rs2280148, the SNP locus in SOCS3 gene, were statistically significant different between the T2DM group and the control group. The "GG" genotype at rs2280148 reduced the risk of T2DM, indicating that the "GG" genotype was resistant to T2DM. It was closely related to T2DM in all three models (*P* < 0.05). Obesity is a risk factor for T2DM. Matthew E Talbert also found that SNPs rs2280148 in SOCS3 gene is closely related to various types of obesity in the American population [26]. Studies have shown that single nucleotide polymorphisms in SOCS3 gene are closely related to obesity and BMI [27, 28]. The above evidences prove that rs2280148 is correlated with T2DM. At the same time, SNP rs2280148 locus is located in the UTR3 of SOCS3, which may be involved in protein transcription and affect the incidence of T2DM. The specific biological mechanism needs further downstream functional verification. The alleles and genotype frequencies of rs10974914 and rs10815157 in JAK2 gene showed statistically significant differences between the diabetic group and the control group. The "AA" genotype at rs10974914 significantly increased the risk of T2DM, while the "C" allele at rs10815157 was the resistance factor for T2DM. Logistic regression analysis showed that two SNPs in JAK2 gene were associated with T2DM in the additive model and recessive model. Zhang [29] found that the overexpression of JAK2 gene could increase the activity of insulin promoter and affect the incidence of T2DM indirectly. There are studies that have reported that Aβ induces hepatic insulin resistance by activating JAK2/STAT3/SOCS-1 signaling pathway and have implications on resolving insulin resistance and T2DM [30]. Therefore, we believe that JAK2 gene may be associated with T2DM in Guangdong Han population and participates in a variety of physiological responses as a member of the JAK/STAT inflammatory signaling pathway.

The analysis results show that haplotypes of S3 (GG) and J3 (GC) may be the protective factors for T2DM, while haplotypes of J2 (AG) and S4 (GT) may be the risk factors for T2DM. However, in STAT3 gene, only one locus in the analysis process met Hardy-Winberg equilibrium test, which did not meet the conditions of haplotype analysis. If forced analysis was carried out, the results would be unstable. After covariate correction, the SNPs-SNPs(rs4969168-rs2280148) interaction of SOCS3 was statistically significant (*OR* = 2.450, *P* < 0.001). These results indicate that there may be a synergistic effect between the two loci of SOCS3 gene, leading to both SNPs loci associated with T2DM and risk factors for T2DM. However, further functional and epigenetic verification is needed to determine the type of interaction between the two SNPs loci. In recessive model, the SNPs rs10815157 interacted with hypertension and was a protective factor of T2DM, indicating that the prevalence of diabetes in hypertensive population with homozygous "C" risk allele was lower than that in hypertensive population without this risk gene. In the recessive model, the SNPs rs1053005 also interacts with hypertension and is a risk factor for T2DM, indicating that hypertensive patients carrying "CC" homozygous gene are more likely to develop diabetes than those without this allele. Martinez et al. [31] showed that SOCS3 gene and hypertension factors jointly act on the population, leading to the onset of T2DM. The SNPs rs4969168 was shown to interact with triglycerides in the additive model, and SNPs rs1053005 was shown to interact with triglycerides in the dominant model. The interaction between the two loci and triglycerides both a risk factor for T2DM. At the same time, the SNPs rs10974914 had an interaction with triglycerides in both additive and recessive models, and this interaction was a factor of resistance to T2DM. Torisu et al. [32] showed that SOCS3 gene and triglyceride could act together to cause the onset of T2DM. The influencing factors of T2DM do not act on individuals alone, but are a combination of multiple factors, and under the action of time effect, genetic factors and environmental factors jointly lead to the occurrence of the disease. The exact mechanism of action is not clear, it may have weak effects on different protein expression and epigenetics aspect, and act on individuals through its combined effect, which is worthy of further study.

## Conclusions

In conclusion, we conducted a preliminary study on the association between SOCS3, JAK2, and STAT3 gene polymorphisms and T2DM in China. The related loci of genes (SOCS3, JAK2) may play a key role in the occurrence and development of T2DM. Gene polymorphisms may provide clues for further study of the pathogenesis of T2DM and search for new drug targets. The different specificity of genetic heterogeneity in T2DM among different populations will be an interesting research direction in the future. Our study used gene haplotype analysis, SNP-SNP interaction analysis, and SNP-environment interaction analysis. We were able to detect moderate and weak susceptibility to T2DM. However, the experiment also has some defects and inadequacies. The sample size and the number of SNPs loci of candidate genes are relatively small, and downstream functional verification has not been verified. As a result, the evidence of biological function is insufficient. At the same time, the association between the six SNPs loci selected in this study and T2DM has rarely been reported. Therefore, a larger sample size, multi-regional, multi-ethnic population study needs to be further carried out.

## Data Availability

The datasets used and/or analysed during the current study are available from the corresponding author on reasonable request.
